# Block of Death-Receptor Apoptosis Protects Mouse Cytomegalovirus from Macrophages and Is a Determinant of Virulence in Immunodeficient Hosts

**DOI:** 10.1371/journal.ppat.1003062

**Published:** 2012-12-13

**Authors:** Linda Ebermann, Zsolt Ruzsics, Carlos A. Guzmán, Nico van Rooijen, Rosaely Casalegno-Garduño, Ulrich Koszinowski, Luka Čičin-Šain

**Affiliations:** 1 Department of Vaccinology and Applied Microbiology, Helmholtz Centre for Infection Research, Braunschweig, Germany; 2 Max von Pettenkofer Institute, Ludwig Maximilians University, Munich, Germany; 3 Department of Molecular Cell Biology, Faculty of Medicine, Vrije University, Amsterdam, The Netherlands; 4 Department of Virology, Hannover School of Medicine, Hannover, Germany; University of Alberta, Canada

## Abstract

The inhibition of death-receptor apoptosis is a conserved viral function. The murine cytomegalovirus (MCMV) gene M36 is a sequence and functional homologue of the human cytomegalovirus gene UL36, and it encodes an inhibitor of apoptosis that binds to caspase-8, blocks downstream signaling and thus contributes to viral fitness in macrophages and *in vivo*. Here we show a direct link between the inability of mutants lacking the M36 gene (ΔM36) to inhibit apoptosis, poor viral growth in macrophage cell cultures and viral *in vivo* fitness and virulence. ΔM36 grew poorly in RAG1 knockout mice and in RAG/IL-2-receptor common gamma chain double knockout mice (RAGγC^−/−^), but the depletion of macrophages in either mouse strain rescued the growth of ΔM36 to almost wild-type levels. This was consistent with the observation that activated macrophages were sufficient to impair ΔM36 growth *in vitro*. Namely, spiking fibroblast cell cultures with activated macrophages had a suppressive effect on ΔM36 growth, which could be reverted by z-VAD-fmk, a chemical apoptosis inhibitor. TNFα from activated macrophages synergized with IFNγ in target cells to inhibit ΔM36 growth. Hence, our data show that poor ΔM36 growth in macrophages does not reflect a defect in tropism, but rather a defect in the suppression of antiviral mediators secreted by macrophages. To the best of our knowledge, this shows for the first time an immune evasion mechanism that protects MCMV selectively from the antiviral activity of macrophages, and thus critically contributes to viral pathogenicity in the immunocompromised host devoid of the adaptive immune system.

## Author Summary

The majority of adult people are infected with human cytomegalovirus (CMV), but in hosts with a healthy immune system it is kept in check and does not cause disease. On the other hand, in patients suffering from innate or acquired immune deficiencies, CMV can cause severe disease or death. Infection of mice with the mouse CMV (MCMV) is an experimental model to study the biology of CMV infection, and mice that lack all of their lymphocytes are very susceptible to MCMV and die typically within three weeks of infection. In this article we show that MCMV causes disease and death in mice lacking lymphocytes because its gene M36 blocks programmed cell death, or apoptosis. MCMV lacking the M36 gene grew thousand folds less well in these mice, which significantly improved survival. This was because M36 deletion made MCMV susceptible to the action of macrophages, cells that secrete soluble factors that induce apoptosis. Importantly, viral growth and virulence of the M36-deficient MCMV could be restored by blocking apoptosis by other means, showing that the block of apoptosis was critical for viral replication. Therefore, our data imply that viral inhibition of apoptosis may be a key molecular target for antiviral strategies in immunodeficient hosts.

## Introduction

The viral inhibition of programmed cell death is a conserved function in animal viruses [1]–[4]. Both autophagy and apoptosis are controlled by most DNA viruses, and frequently this is done by redundant gene products. The herpes simplex gene ICP34.5 and the cytomegalovirus (CMV) gene TRS1 both bind the host gene Beclin and thus block autophagy [5], [6]. Similarly, both the human CMV (HCMV) and the murine CMV (MCMV) encode viral genes that block mitochondrial apoptosis [7], [8] by blocking Bax, but not Bak signaling [7], [9]. The HCMV gene has been termed viral Inhibitor of Mitochondrial Apoptosis (vMIA), and corresponds to exon 1 of the viral gene UL37 (UL37x1), whereas M38.5 is the MCMV counterpart. While the two genes show no sequence homology, they occupy essentially the same position in the viral genome and exert the same function at the molecular level, arguing for the importance of this function. An even more remarkable conservation of gene function has been observed in the case of the viral Inhibitor of Caspase-8 Activation (vICA), encoded by the HCMV gene UL36, and its counterpart in the MCMV genome, a gene called M36 [10], [11]. The UL36 and M36 genes show conserved sequence homology, and both block death-receptor apoptosis by binding to caspase-8 [10], [11]. Interestingly, MCMV lacking the M36 gene (ΔM36) could not grow to high titers in macrophage cell lines or in primary macrophages, but showed no loss of fitness in fibroblasts [10], which is in line with the observations that UL36 is required for HCMV growth in macrophages derived from THP-1 monocytes [12], but not for its growth in fibroblasts [13].

Experiments in tissue culture systems and *in vivo* demonstrated that the inhibition of apoptosis by viral gene products is a determinant of viral fitness [12], [14], [15]. In the case of the UL36 gene, it has been shown that the inhibition of caspase signaling by the pan-caspase inhibitor carbobenzoxy-valyl-alanyl-aspartyl-[O-methyl]- fluoromethylketone (z-VAD-fmk) restores growth of deletion mutants in immature, but not in mature macrophages [12], indicating that apoptosis limits the replication of the UL36-deficient virus in defined cell types. On the other hand, z-VAD-fmk completely rescued the growth of the M36-deficient mutant in murine macrophages [14]. We previously showed that the lack of the viral gene M36 results also in severe growth deficits *in vivo* [14], [16], which could be rescued by the replacement of the M36 gene with a dominant-negative variant of the Fas-associated via Death Domain (FADD^DN^) gene, inserted into the viral genome [14]. Therefore, apoptosis was shown to be a determinant of MCMV fitness in macrophages and *in vivo*, arguing that the viral replication in macrophages might be a key determinant of viral fitness, but a direct demonstration for this assertion remained pending. Moreover, it remained unclear if viral inhibition of apoptosis plays a role in viral fitness in the immunocompromised host, or merely in the immunocompetent one. This question may be of key clinical relevance, as CMV infection results in overt disease in the immunocompromised and rarely in the immunocompetent host. Finally, it remained unclear why the inhibition of caspase-8 plays an important role in viral fitness in macrophages, but not in other cell types.

We show here that the M36-mediated block of apoptosis is not a determinant of viral tropism for macrophages, but rather a function enabling viral growth by protecting infected cells from macrophage-secreted TNFα. Moreover we show that the viral inhibition of death-receptor apoptosis is a key determinant of viral growth and virulence in the immunodeficient host. Finally, we show that macrophages protect the immunodeficient host from a virus mutant lacking the M36 gene. Since the inhibition of death-receptor signaling is a molecular mechanism that is conserved in all CMV species, we define here the viral inhibition of apoptosis as a key molecular target for the development of novel antiviral strategies protecting specifically the immunodeficient host from CMV disease.

## Results

### M36 Is a Determinant of Virulence in RAG1 Knockout Mice

**Figure 1:**
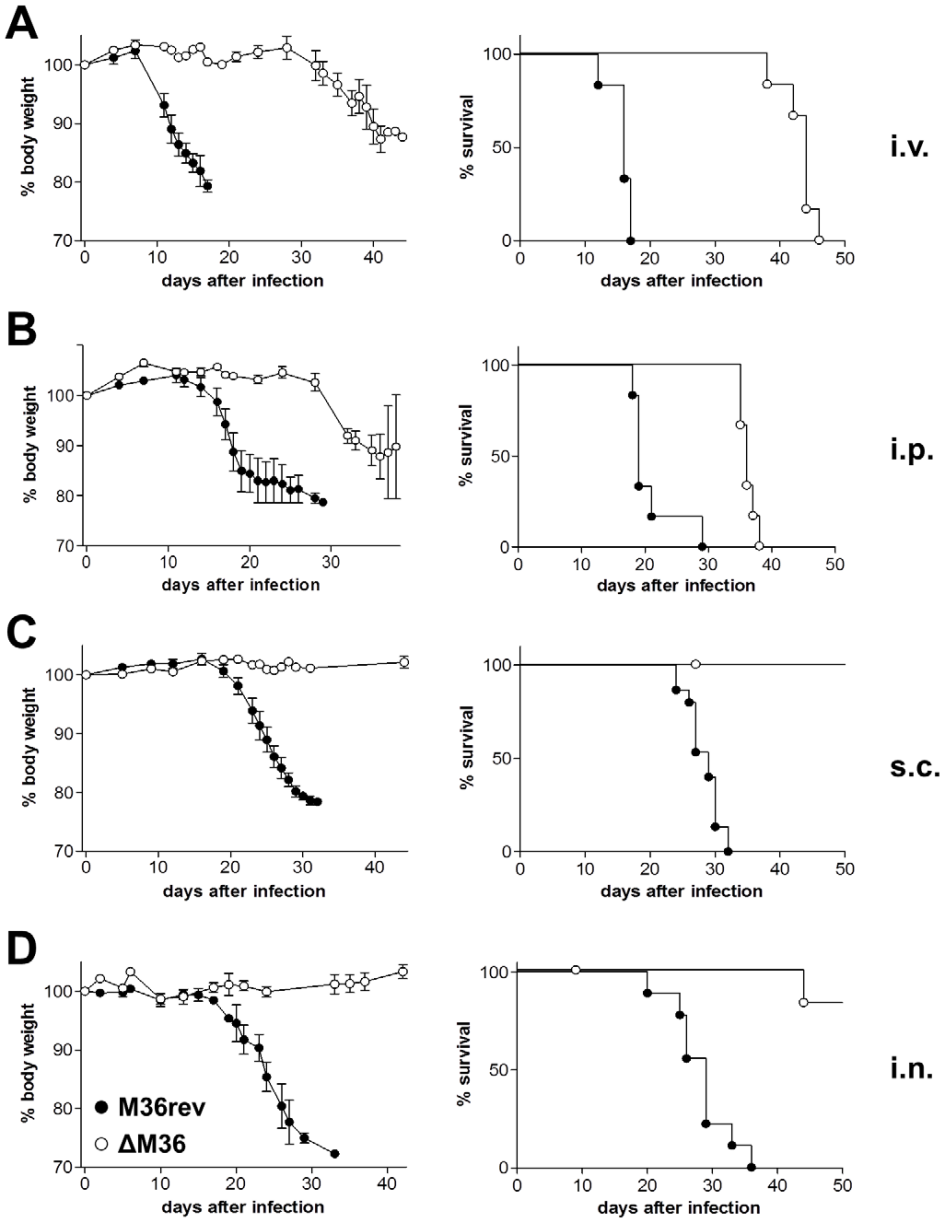
ΔM36 MCMV applied locally is avirulent in RAG1^−/−^ mice. RAG1^−/−^ mice were infected by (A) intravenous, (B) intraperitoneal, (C) subcutaneous, or (D) intranasal administration with 10^5^ PFU of ΔM36 (○) or M36rev (•) MCMV (n = 6–15/group) and monitored for weight loss and survival. Mortality also includes mice that were sacrificed because they had lost more than 20% of body weight.

We showed previously that the inhibition of death-receptor apoptosis by the M36 gene is necessary for MCMV growth in multiple organs of mice, and is particularly important for viral growth in the salivary glands [14]. It remained unclear whether M36 is also a determinant of virulence. Cytomegalovirus causes clinical symptoms in immunodeficient hosts; therefore, an experimental model of CMV pathogenicity requires the infection of immunodeficient mice. We tested ΔM36 virulence in recombinase activating gene 1 (RAG1) knockout mice, which are deficient for B and T lymphocytes. The cytotoxic activity of CD8 T-cells and the secretion of TNFα by activated CD8 and/or CD4 T-cells have both the potential to induce death-receptor apoptosis in virus-infected cells. We reasoned that the lack of these functions in RAG1^−/−^ mice may contribute to their inability to control viral growth. In that case, the growth and virulence of ΔM36 MCMV, which is unable to block death receptor apoptosis, would be rescued in RAG1^−/−^ mice. Upon intravenous (i.v.) infection, we observed an unexpected delay in the loss of body weight and subsequent mortality of ΔM36 infected mice (Figure 1A). The same observation could be made in intraperitoneally (i.p.) infected mice (Figure 1B). Even more remarkably, subcutaneous (s.c.) or intranasal (i.n.) infection with ΔM36 resulted in no significant morbidity or mortality up to 4 months post infection, whereas all mice infected with the control virus M36rev MCMV died by day 40 post infection (Figure 1C, 1D). Therefore, while systemic administration of ΔM36 resulted in a delayed mortality compared to the revertant virus, mucosal or local administration resulted in significantly lower mortality and morbidity of mice infected with the same virus.

### M36-Mediated Block of Apoptosis Is a Determinant of Viral Fitness and Virulence in RAG1 Knockout Mice

**Figure 2:**
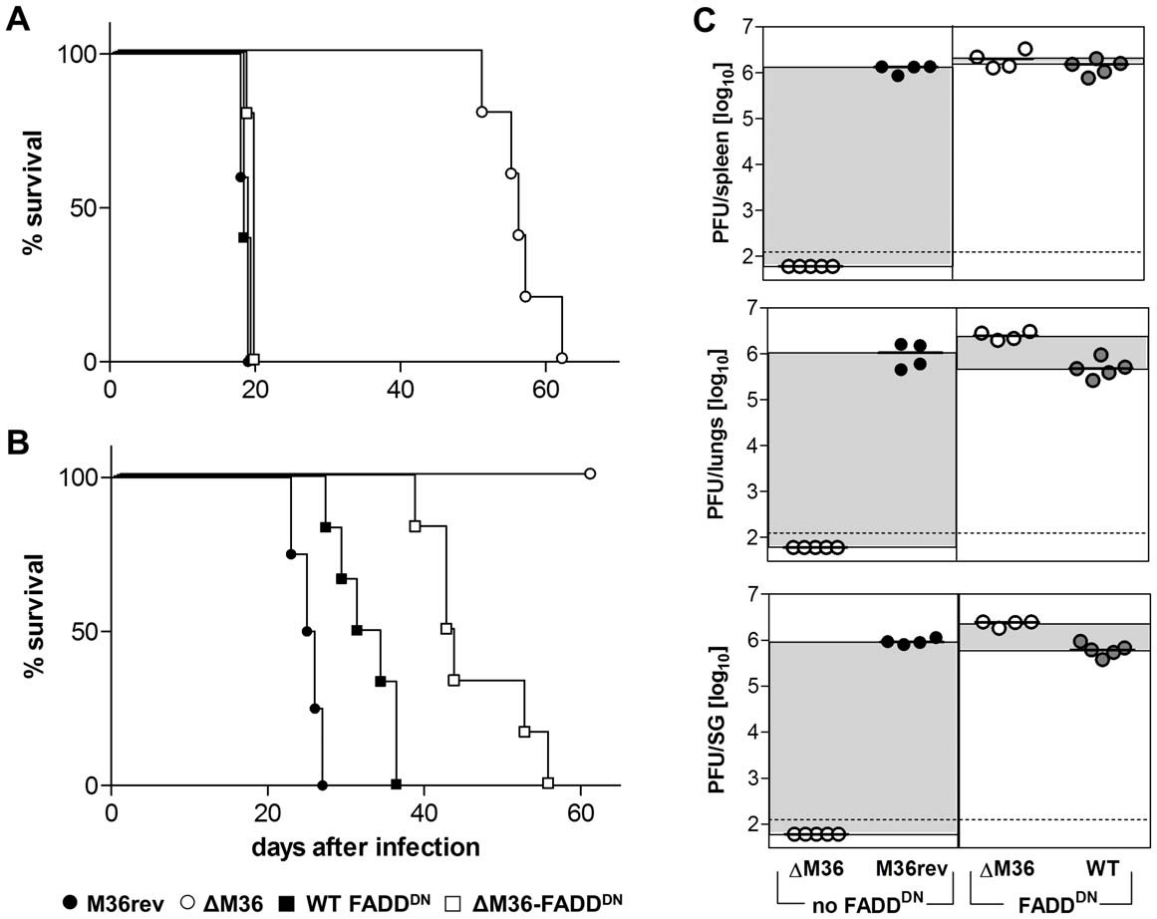
Apoptosis inhibition is required for viral dissemination to distant organs. RAG1^−/−^ mice were (A) i.p. or (B) s.c. infected with 10^5^ PFU of indicated virus and monitored for survival (n = 4–6/group). Mortality also includes mice that were sacrificed because they had lost more than 20% of body weight. (C) Infectious virus was determined by plaque assay on MEF cells in spleen (top panel), lungs (middle panel), and salivary glands (SG, bottom panel) of i.p. infected mice on day 13 after infection with 10^5^ PFU of indicated virus. Each symbol represents an individual mouse. Differences in median values are highlighted by grey shading. The dashed line shows the limit of detection.

The M36 gene is an inhibitor of death-receptor mediated apoptosis [10], and we showed that the block of apoptosis is a determinant of viral *in vivo* fitness [14]. Therefore, it was conceivable that the loss of virulence was due to poor viral replication caused by an increase in apoptosis. This was tested by infecting mice with a recombinant lacking the M36 gene, but carrying a dominant-negative FADD gene (ΔM36-FADD^DN^), which replaced the anti-apoptotic function of M36 in cells infected with the recombinant virus. Mice infected with the viruses expressing the FADD^DN^ gene died essentially at the same time as the M36rev infected mice when the infection was performed i.p. (Figure 2A) and with a moderate delay upon s.c. infection (Figure 2B), yet even ΔM36-FADD^DN^ mice showed 100% mortality upon infection. At day 13 post infection, we observed a complete rescue of the virus titer in spleen, lungs, and salivary glands (Figure 2C), strongly arguing that the inhibition of death-receptor apoptosis is required for MCMV replication in RAG1^−/−^ mice, and MCMV virulence in immunocompromised hosts. Analysis of virus titer in various organs at 1, 2 or 3 months post s.c. infection confirmed that ΔM36 MCMV is not present in the lungs or salivary glands after approximately four weeks, but also revealed that low levels of replicative virus are still detectable in the spleen of some ΔM36 MCMV infected mice for up to 90 days post infection (Figure S1).

### M36 Promotes Viral Growth in Mice Lacking B, T and NK Cells

**Figure 3:**
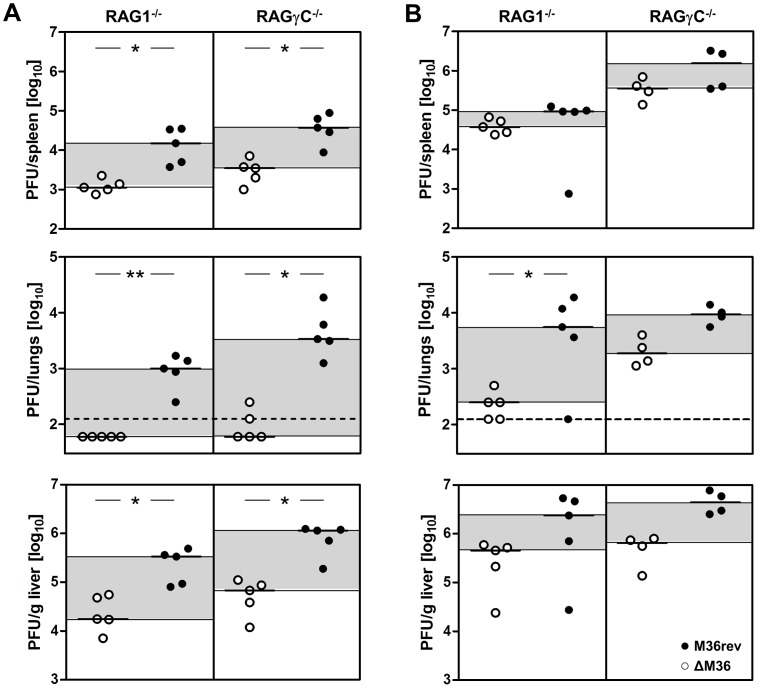
Macrophage, but not NK cell, depletion rescues ΔM36 MCMV *in vivo*. In a combined experiment to elucidate the role of (A) NK cells and (B) macrophages in the control of ΔM36 MCMV growth, RAG1^−/−^ and RAGγC^−/−^ mice received injections of 200 µl liposome encapsulated (A) PBS or (B) clodronate 48 hours (i.v.) and 24 hours (i.p.) prior to viral infection. Following liposome injection mice were i.p. injected with 10^5^ PFU ΔM36 (○) or M36rev (•) MCMV (n = 4–5/group). At day 3 post infection infectious virus was determined by plaque assay on MEF cells in spleen (top panels), lungs (middle panels) and liver (bottom panels). Each symbol represents an individual mouse. Differences in median values are highlighted by grey shading. The dashed line shows the limit of detection. *p<0.05; **p<0.01.

Death-receptor apoptosis inhibited ΔM36 growth in RAG1^−/−^ mice (Figure 2C). Since these mice lack cytotoxic T-cells, we assumed that another cell type may inhibit ΔM36 growth by inducing apoptosis of ΔM36 infected cells through the death-receptor. We considered the possibility that T-cells and NK cells may control the virus in a redundant fashion and therefore infected mice lacking B, T and NK cells due to targeted deletions of the IL-2-receptor common gamma chain and of the RAG2 gene (RAGγC^−/−^ mice). Three days following i.p. injection the infectious ΔM36 titer was marginally higher in the spleen, liver, and lungs of RAGγC^−/−^ mice than in the organs of RAG1^−/−^ mice, but this increase was not specific for the mutant virus, because we observed a similar increase in the titer of M36rev MCMV (Figure 3A). This was in line with our observations that RAGγC^−/−^ mice survived much longer when infected with ΔM36 MCMV than with M36rev (Figure S2A), in accordance with our results from RAG1^−/−^ mice. The results were essentially identical in RAG1^−/−^ mice depleted of NK cells by continuous treatment with anti-Asialo-GM1 antibodies before and after infection with MCMV viruses (Figure S2B). Therefore, our data indicated that NK cells were not crucial in the control of ΔM36 *in vivo* growth, but rather that other cells of the innate immune system contributed to the control of ΔM36 replication through a death-receptor mediated mechanism.

### Macrophage Depletion Rescues ΔM36 Growth in RAG1^−/−^ and RAGγC^−/−^ Mice

We next considered that macrophages may be important in the control of ΔM36 growth, because it has been shown that ΔM36 MCMV has a macrophage-specific growth defect [10]. Therefore, we depleted macrophages in RAG1^−/−^ and RAGγC^−/−^ mice by i.p. injection of liposome encapsulated clodronate [17], [18]. A strong but not complete macrophage depletion was confirmed by flow cytometry in spleen and the peritoneal cavity, whereas the depletion was less efficient in the liver (Figure S3). We could observe that macrophage depletion rescued ΔM36 growth in spleen and lungs, almost to levels seen in mice infected with the revertant virus whereas the rescue was not as pronounced in the liver (Figure 3B), consistent with the less efficient macrophage depletion. Therefore, our data indicated that M36 allows MCMV growth in the presence of macrophages.

### Zymosan and IFNγ Impair ΔM36 Growth in Primary MEF, but Not in 3T3 Fibroblasts

**Figure 4:**
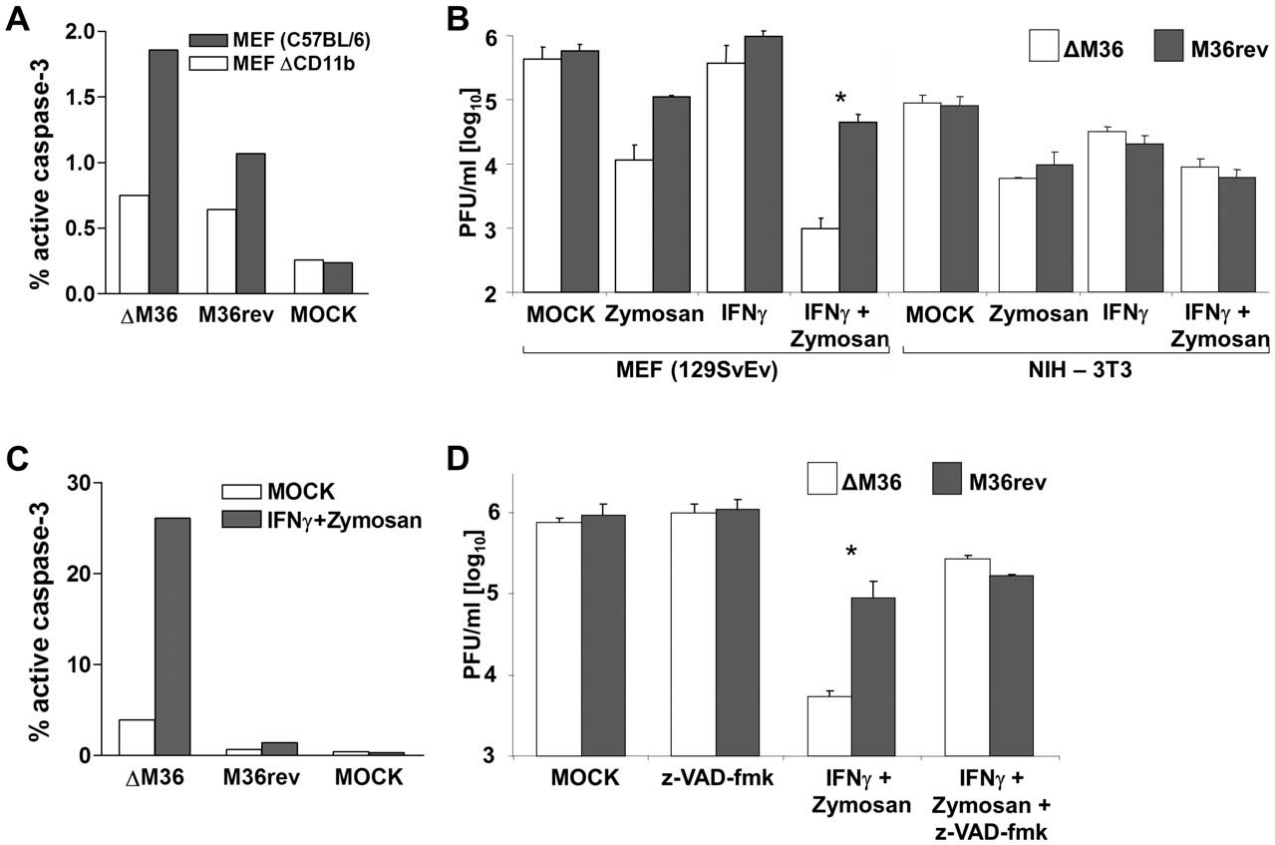
Apoptosis of primary MEF cells inhibits ΔM36 growth. (A) MEF cells and MEF preparations depleted of CD11b positive cells (MEF ΔCD11b) were infected with indicated virus at a MOI of 1, and 24 hours later analyzed for the induction of the active isoform of caspase-3 by flow cytometry. The percentage of caspase-3 positive cells in a representative experiment is indicated. (B) MEF or NIH-3T3 cells were infected at a MOI of 0.03 with ΔM36 (white bars) or M36rev (grey bars) and supernatants were titrated for infectious MCMV titer at day 4 post infection. Where indicated, Zymosan (30 µg/ml) and/or IFNγ (100 ng/ml) were added to the supernatant immediately following infection. Histograms indicate means from three experiments, error bars are standard deviations. (C) MEF cells were infected at a MOI of 5 with ΔM36, M36rev or mock-infected. Where indicated, IFNγ (100 ng/ml) and Zymosan (30 µg/ml) were added to the supernatant immediately following infection. Expression of the active isoform of caspase-3 was measured 24 hours post infection by flow cytometry and the percentage of caspase-3 positive cells in the total cell pool is indicated. (D) MEF cells were infected at a MOI of 0.03 with ΔM36 (white bars) or M36rev (grey bars) and supernatants were titrated for infectious MCMV at day 4 post infection. Where indicated, z-VAD-fmk (33 µM), Zymosan (30 µg/ml) and IFNγ (100 ng/ml) were added to the supernatant immediately following infection. Histograms indicate means from three experiments, error bars are standard deviations, * p<0.05.

To study the mechanism by which macrophages limit ΔM36 MCMV growth, we turned to cell culture conditions that contained mixtures of macrophages and other cell types. Preparations of primary mouse embryonic fibroblast (MEF) cells consistently contain a fraction of CD11b positive cells which are absent from 3T3 fibroblast cell lines (Figure S4). Testing the induction of apoptosis in MEF cells infected with ΔM36 MCMV, resulted in a significant increase in the fraction of cells expressing the activated isoform of caspase-3, a marker of apoptosis, over the M36rev- or mock-infected controls, which was dependent on the presence of CD11b positive cells (Figure 4A). This was in contrast with our previous observations that fibroblast cell lines do not undergo apoptosis upon ΔM36 infection, unless stimulated with anti-FAS antibodies [10]. Since ΔM36 induced apoptosis in primary MEF cells, we compared ΔM36 to M36rev MCMV growth in primary MEF cells and NIH-3T3 fibroblasts. To test if macrophages need to be activated to control the virus growth, we activated them by administering IFNγ, Zymosan (a toll-like receptor 2 agonist), or both of these mediators in combination to the infected cells. We observed no significant difference in ΔM36 and M36rev titers when the cells were infected in absence of stimuli which may activate macrophages, but a substantial reduction of ΔM36 titer in the presence of IFNγ and Zymosan. The combination of IFNγ and Zymosan resulted in a 100-fold reduction of ΔM36 proliferation in MEF cells, but had no effect in NIH-3T3 cells (Figure 4B). This was consistent with a dramatic increase in apoptosis of ΔM36 infected cells upon combined treatment with IFNγ and Zymosan (Figure 4C). Similarly, addition of LPS and IFNγ suppressed ΔM36 growth to the same extent, indicating that the antiviral action was not exclusive to TLR2 activation (Figure S5). Interestingly, the reduction in virus titer could only be observed when cells were infected at a multiplicity of infection (MOI) below 0.1, but not at higher MOIs (data not shown). Z-VAD-fmk, a pan-caspase inhibitor, rescued ΔM36 growth in MEF cells treated with Zymosan and IFNγ to levels seen in M36rev (Figure 4D), arguing strongly that M36 improved MCMV dissemination on MEF cells by blocking caspase signaling, which would be in line with the *in vivo* data (Figure 2).

### Macrophage Contaminants Impair ΔM36 Growth in Primary MEF Cells

**Figure 5:**
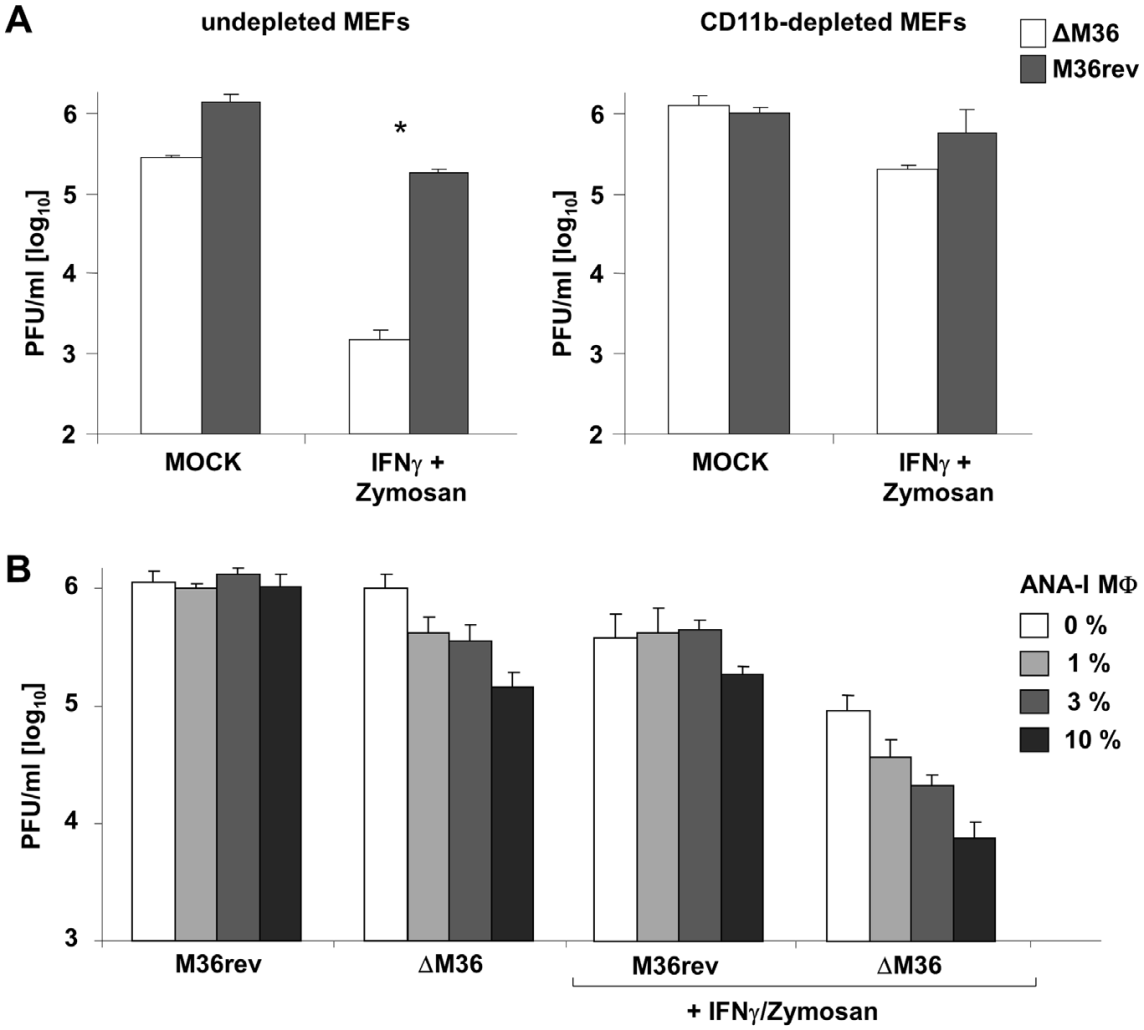
ΔM36 grows poorly in the presence of macrophages. (A) CD11b positive cells were removed from MEF cell preparations by monoclonal antibodies coupled to magnetic beads, upon which the cells were infected with ΔM36 (white bars) or M36rev (grey bars), alone or in the presence of Zymosan (30 µg/ml) or IFNγ (100 ng/ml). Virus titer in the supernatant of cells depleted of macrophages was compared to macrophage-undepleted MEF preparations at day 4 post infection. (B) Upon macrophage depletion, primary fibroblasts were cultured with indicated amounts of ANA-I macrophages (MΦ), in the presence or absence of Zymosan (30 µg/ml) and IFNγ (100 ng/ml). Infectious virus titer in supernatants was established at day 4 post infection. Histograms indicate mean values from three separate experiments, error bars show standard deviation, * p<0.05.

To directly test the role of macrophages in ΔM36 growth, we depleted the CD11b positive cells from MEF cell preparations by cell separation using anti—CD11b antibodies coupled to magnetic beads and collected the cells from the unbound fraction. The efficiency of the depletion was confirmed by flow cytometry for CD11b, upon which the cells were infected and ΔM36 growth was compared to M36rev in presence or absence of Zymosan and IFNγ. CD11b depletion resulted in a complete rescue of ΔM36 growth, even in the presence of Zymosan and IFNγ (Figure 5A), demonstrating that the poor growth was not due to intrinsic differences between primary fibroblasts and fibroblast cell lines, and strongly suggesting that CD11b positive cells are required for the inhibition of ΔM36 growth.

To confirm that macrophages were necessary and sufficient to control ΔM36 growth in a milieu containing Zymosan and IFNγ, we cocultivated the CD11b-depleted MEF cells with increasing amounts of ANA-I macrophages, a macrophage cell line. We could readily observe a suppression of ΔM36, but not M36rev growth that was dependent on the percentage of macrophages in cell culture (Figure 5B). While ANA-I cells at higher concentrations suppressed ΔM36 growth even in the absence of Zymosan and IFNγ, the effect was more pronounced in their presence (Figure 5B), consistent with our earlier data (Figure 4B). Therefore, our data strongly argued that macrophages control the growth of ΔM36 in mixed populations of cells.

### IFNγ Impairs ΔM36 Growth in Macrophage/Fibroblasts Cocultures by Acting on Fibroblasts and Not on Macrophages

**Figure 6:**
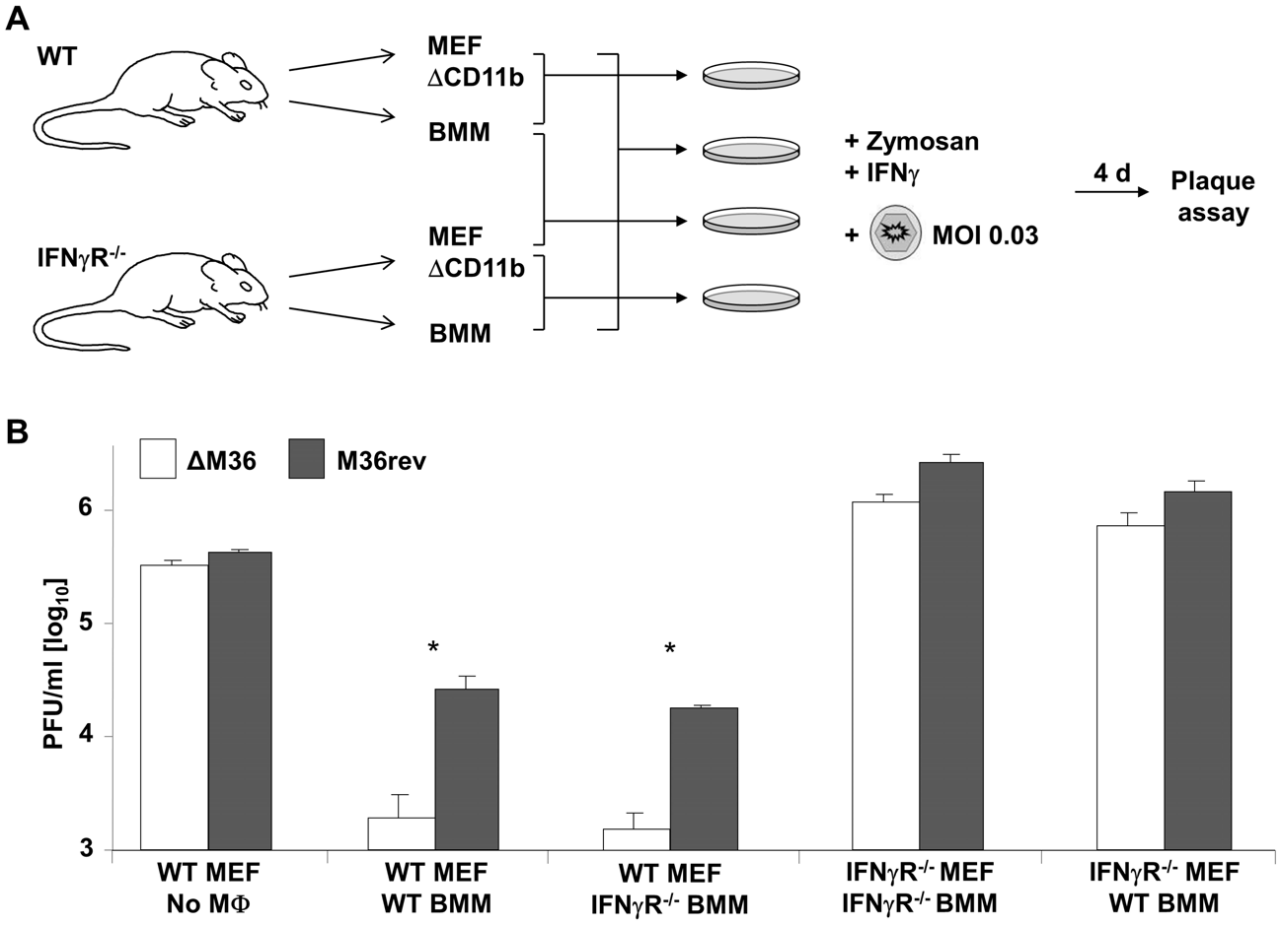
IFNγ controls ΔM36 growth by acting on the IFNγ receptor on fibroblasts, not on macrophages. (A) Experimental setup: MEF cells depleted for CD11b positive cells (MEF ΔCD11b) and BM-derived macrophages (BMM) were obtained from IFNγRec^−/−^ (IFNγR^−/−^) or wild-type (WT) mice and cocultured (10% of macrophages, 90% of fibroblasts in cell culture) in all possible combinations. Cells were infected with ΔM36 or M36rev in the presence of Zymosan (30 µg/ml) and IFNγ (100 ng/ml) and virus titer in the supernatants was established at day 4 post infection. (B) Infectious titer of ΔM36 (white bars) or M36rev MCMV (grey bars) is shown as mean+standard deviation from three independent experiments. The combination of cells used in the infectious experiment is indicated below the x-axis, * p<0.05.

The IFN type II receptor is present on both fibroblasts and macrophages. Therefore, it was possible that IFNγ suppressed ΔM36 growth by acting on macrophages or on fibroblasts. To define which scenario occurred, we prepared CD11b-depleted cultures of primary MEF cells from mice lacking the IFN type II receptor (IFNγRec^−/−^), or from the parental mouse strain, and cocultivated the fibroblasts with bone marrow (BM)-derived macrophages derived from IFNγRec^−/−^ mice or BM-derived macrophages derived from the parental mouse strain (Figure 6A). We observed a difference in the titers of ΔM36 and M36rev regardless of the source of macrophages used in the cocultivation assay (Figure 6B). On the other hand, ΔM36 growth was rescued to revertant-levels in macrophage-depleted MEF cells from IFNγRec^−/−^ mice, and here it was irrelevant whether we added macrophages from IFNγRec^−/−^ mice or the parental mouse strain (Figure 6B). Hence, our data showed that IFNγ controlled ΔM36 growth by acting on fibroblasts and not on macrophages.

### TNFα Secreted from Activated Macrophages Impairs ΔM36 Replication in Fibroblasts through a Caspase-Dependent Mechanism

**Figure 7:**
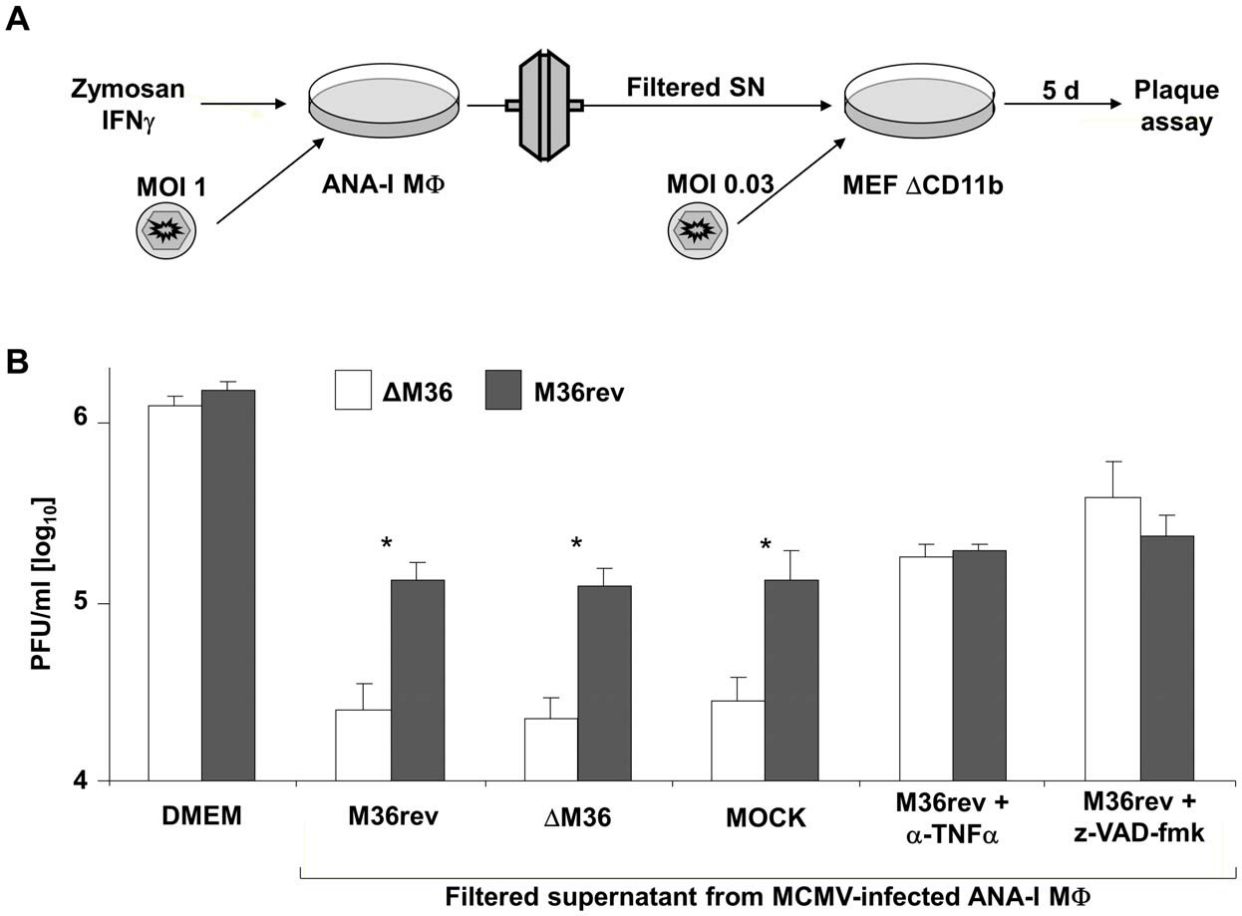
TNFα secreted from macrophages synergizes with IFNγ to impair ΔM36 growth by a caspase-dependent mechanism. (A) Experimental setup: ANA-I cells were treated for 5 days with Zymosan and IFNγ in the presence of ΔM36 MCMV, M36rev MCMV, or no virus, upon which the supernatants were filtered to prevent virus carryover and transferred to CD11b-depleted MEF cells infected with ΔM36 or M36rev. (B) Infectious titer of ΔM36 (white bars) or M36rev-MCMV (grey bars) at 5 days post infection. Legends below the x-axis indicate the medium used during infection – control medium (DMEM), supernatant from ANA-I cells infected with ΔM36, M36rev or no virus (MOCK). Where indicated, the ANA-I supernatant was supplemented with neutralizing anti-TNFα (1 µg/ml) antibodies or z-VAD-fmk (33 µM). Histograms indicate mean values from three separate experiments, error bars show SD, * p<0.05.

The mechanism by which macrophages blocked the growth of ΔM36 in MEF cell preparations remained unclear. Macrophage depletion did not result in a loss of M36rev titer, showing that viral replication in the macrophage population was not a dominant factor. Therefore, we hypothesized that activated macrophages may block the growth of ΔM36 by releasing factors that inhibit ΔM36 growth in fibroblasts. To test this, we treated ANA-I macrophages with IFNγ and Zymosan for 5 days and transferred their supernatants on CD11b-depleted MEF cells infected with ΔM36 or M36rev (Figure 7A). To elucidate the contribution of the virus infection to the macrophage activation, we infected the macrophages at a MOI of 1 with ΔM36, M36rev or mock-infected them in the presence of Zymosan and IFNγ. Supernatants were filtered using a 100 nm filter to prevent carryover of virus from ANA-I supernatants into MEF cultures (Figure 7A). We observed ΔM36 growth reduction in cells treated with ANA-I derived supernatant, but not in control cells grown in normal medium (Figure 7B). Moreover, we could see that it was irrelevant if the macrophage cultures were infected with virus or not, showing that the impact of the ANA-I supernatant on ΔM36 growth was due to effects on fibroblasts (Figure 7B). Z-VAD-fmk treatment of MEF cells promptly rescued the ΔM36 growth to WT levels, indicating that the mechanism of action was caspase-dependent (Figure 7B). Since TNFα is a factor that is secreted from activated macrophages, and has the ability to induce apoptosis in target cells by a caspase-8 dependent signaling pathway, we considered the possibility that macrophages control ΔM36 by secreting TNFα. Therefore, we added neutralizing antibodies against TNFα to the ANA-I supernatant used in wells infected with ΔM36 or M36rev. We observed that anti-TNFα antibodies rescued the growth of ΔM36 in fibroblasts (Figure 7B). Therefore, our data strongly indicated that TNFα secreted from macrophages is necessary to control ΔM36 growth in fibroblasts.

## Discussion

*In vitro* ΔM36 MCMV growth is known to be attenuated in macrophages, which was due to viral inhibition of caspase signaling, and appeared to be particularly dependent on the death-receptor signaling pathway [10], [14]. Similarly, the mutant was attenuated *in vivo* [16] due to poor viral inhibition of death-receptor signaling [14]. However, it remained unclear if the viral inability to grow in macrophages contributed to its *in vivo* attenuation. Poor ΔM36 *in vivo* growth was related to its macrophage phenotype, but surprisingly, not as a result of a defect in tropism for macrophages. Instead we observed that ΔM36 MCMV does not only grow poorly in macrophage populations, but also in their mere presence. In fact, ΔM36 grew poorly because it could not counter the antiviral factors secreted by macrophages. While our results do not formally exclude the possibility that M36 additionally acts as a tropism factor in macrophages, for instance by functions unrelated to apoptosis, our data argue strongly that the inability of a CMV variant to grow in a cell type does not necessarily reflect a lack of tropism. This finding might bear relevance for other viruses where tropism has been experimentally validated by the virus ability to grow in pure cultures of a defined type. For instance, the tropism of varicella-zoster virus for T-cells or adenovirus tropism for hematopoietic cells have both been linked to viral genes that inhibit apoptosis [4], [19]. We postulate here that the secretion of death-receptor ligands from infected cells may act in a paracrine fashion and in a cell-type unrestricted manner. Therefore, the viral inhibition of the death receptor pathway may mask as a determinant of tropism, while reflecting an immune evasion mechanism that rescues the virus from the antiviral action of cells of a specific type. Future studies will clarify to which extent this observation can be generalized to other viruses.

It has been previously shown that M45, another MCMV-encoded anti-apoptotic gene [20], is necessary for viral growth in immunodeficient SCID mice [21]. However, it remained unclear if this was due to the anti-apoptotic function of the M45 gene, or to some other property encoded by the same gene, for instance its ability to block NF-κB signaling [22]. We show here that the pathogenicity of MCMVs expressing M36 is directly related to their ability to block death-receptor apoptosis, because the virulence and the viral replication in the immunodeficient host could be rescued by the replacement of M36 with another gene blocking the same signaling cascade.

McCormick *et al*. recently showed that the HCMV gene UL36 blocks apoptotic and non-apoptotic cell death in mature THP1 cells infected with HCMV [12]. While our data showed a clear role for the M36-mediated inhibition of apoptosis, our results cannot formally exclude the possibility that M36 may also affect other functions important for viral replication. This would be particularly important in line of recent observations that caspase-8 signaling suppresses RIP1/RIP3-dependent necrotic or necroptotic cell death pathways [23] and that the suppression of this pathway by the MCMV gene M45 [24], [25] plays a crucial role in viral *in vivo* growth [24]. Hence, the modulation of caspase-8 signaling by M36 may also have effects on RIP1/RIP3-dependent pathways, and it remains unclear if the M36-mediated block of the caspase-8 dependent apoptotic pathway results is a similar block of caspase-8 effects on non-apoptotic cell death. Therefore, more work is needed to clarify the role of M36/UL36 on the RIP1/RIP3 pathway.

It has been shown that macrophages play an important role in the control of *in vivo* MCMV replication [26], [27], yet the exact mechanism of their antiviral action remained unclear. We show here that apoptosis plays an important role in the antiviral macrophage activity, because mice that lacked both NK and T-cells but contained macrophages controlled ΔM36 MCMV much more efficiently than the revertant, but macrophage depletion rescued ΔM36 in these mice to titers that almost matched those seen in the infection with the revertant virus. While our data do not exclude the possibility that ΔM36 was controlled by redundant antiviral activity from NK, CD8 T-cells and macrophages, they clearly indicated that macrophages were sufficient for the *in vivo* control of ΔM36. Conversely, M36 was necessary for viral replication and virulence in the immunocompromised host lacking T-cells or NK and T-cells, because it allowed the virus to overcome the antiviral activity of macrophages. This finding may also have clinically relevant consequences, because most CMV-related disease occurs in immunocompromised patients and not in the immunocompetent ones. Therefore, the inhibition of death-receptor apoptosis might be a virulence factor that would affect CMV pathogenesis specifically in the absence of a mature or functional adaptive immune system.

Macrophage depletion in mice did not result in a complete rescue of ΔM36 growth to titers seen upon infection with the revertant. This result may indicate that there may be additional cell types which also contribute to the selective *in vivo* control of ΔM36 MCMV growth, or it may be due to the fact that the macrophage depletion was not 100% efficient, and that the small fraction of macrophages spared from the treatment with liposome encapsulated clodronate was sufficient to affect selectively ΔM36 growth. This is both consistent with observations that treatment with liposome encapsulated clodronate does not allow for a complete depletion of macrophages resident in solid organs [28], and our observations that a fraction of 1% of macrophages could affect the growth of ΔM36 in tissue culture (Figure 5B).

**Figure 8:**
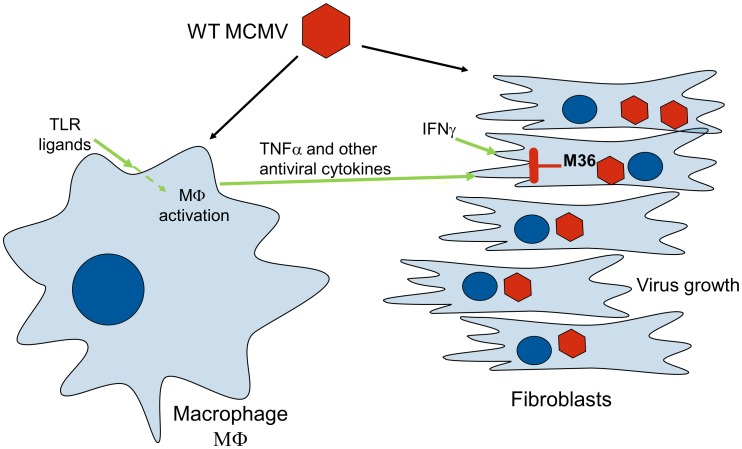
Diagram of the proposed mechanism of action. Activated macrophages secrete TNFα (and possibly additional cytokines) which synergize with IFNγ in fibroblasts to block virus growth by a mechanism that is dependent on caspase signaling. M36 blocks the caspase-dependent signaling pathway and thus prevents apoptosis and rescues the virus growth.

Interestingly, we could observe that the *in vitro* antiviral activity of macrophages was entirely dependent on a costimulation with IFNγ (Figure 4B, Figure 6). Hence, TNFα was necessary, but not sufficient, to control viral growth *in vitro*. We showed previously that TNFα is neither necessary, nor sufficient to control ΔM36 MCMV *in vivo*, because the mutant remained attenuated in TNFR KO mice [14]. The difference between our current *in vitro* evidence, and the former *in vivo* results, is consistent with a model where TNFα, Fas-ligand or the Trail-ligand activate the death-receptors in a redundant fashion, but merge at the FADD level, which is also in line with the observation that ΔM36 could not be rescued in FAS^−/−^ or TNFRp55^−/−^ mice, but was rescued by FADD^DN^ [14]. While the synergic anti-CMV activity of TNFα and IFNγ is well described in literature [29], [30], it was not clear, to the best of our knowledge, that this activity critically depends on caspase signaling. It remains unclear if M36 may also contribute to the protection of the virus from cytokines secreted by NK cells, CD8 T-cells or Th1 CD4 T-cells. Our data would suggest that M36 may protect the virus against multiple effector mechanisms. We propose a model of action, where activated macrophages secrete TNFα which synergizes with IFNγ in fibroblasts to block virus growth, yet M36, by blocking a caspase-dependent mechanism prevents apoptosis and rescues the virus growth (Figure 8). We demonstrated that macrophages control specifically ΔM36 growth *in vivo* and that the viral *in vivo* growth and virulence was critically dependent on death-receptor signaling. Therefore, we postulate that M36/UL36 may present excellent targets for antiviral therapies.

## Materials and Methods

### Ethics Statement

All animal experiments were performed in compliance with the German animal protection law (TierSchG BGBl. S. 1105; 25.05.1998). The mice were housed and handled in accordance with good animal practice as defined by FELASA and the national animal welfare body GV-SOLAS. All animal experiments were approved by the responsible state office (Lower Saxony State Office of Consumer Protection and Food Safety) under permit number 33.9-42502-04-11/0426.

### Mice

RAG1^−/−^ and RAGγC^−/−^ mice were bred at the Helmholtz Centre for Infection Research (Braunschweig, Germany). BALB/c mice were purchased from Janvier (Le Genest Saint Isle, France).

### Cells

M2-10B4 (ATCC; catalog no. CRL-1972) cells were maintained in Dulbecco's modified minimal essential medium (DMEM) supplemented with 10% fetal calf serum (FCS), L-glutamine and penicillin/streptomycin. ANA-I and NIH-3T3 cells were grown as described previously [14]. C57BL/6, 129SV2 and IFNγRec^−/−^ (on an 129SV2 background) murine embryonic fibroblasts (MEFs) were prepared and maintained as described previously [31]. Primary bone-marrow macrophages were isolated and grown by standard procedures, essentially as described elsewhere. In brief, cells from the cavities of both tibial bones were flushed in 5 ml PBS, briefly spun down and resuspended by pipetting them up and down in DMEM supplemented with 10% FCS. One quarter of total cells from one mouse was layered in a 10-cm Petri dish, in medium supplemented with 10 ng/ml of recombinant murine macrophage-colony stimulating factor (M-CSF, Sigma, catalog no. M9170). Two days later, the non-adherent cells were discarded, and medium with fresh M-CSF was added. Macrophages were harvested 7 days post isolation and used in experiments as detailed elsewhere.

### Viruses

The viruses used in this study, MCMV-M36rev (briefly M36rev), MCMV-ΔM36 (ΔM36) and FADD^DN^ overexpressing viruses, have been described previously by Cicin-Sain *et al*. [14] and Menard *et al*. [10]. Virus stocks were generated on M2-10B4 cells and quantified by plaque assay on MEFs as described previously [10], [31].

### Flow Cytometry

MEF cells from a 6-well plate were harvested by trypsination and stained for 30 minutes with a 1∶40 dilution of purified α-CD16/32 antibodies to block the Fc receptor and avoid unspecific staining. Following that, cells were stained with a 1∶100 dilution of α-CD11b-TRICOLOR (Invitrogen) or α-CD11c-PE (Invitrogen) antibodies in 100 µl PBS, 2% FCS, upon which the cells were washed in 5 ml of PBS and analyzed in an EPICS XL flow cytometer (Beckmann Coulter). MEF cells were stained for the active isoform of caspase-3 exactly as previously described [14].

### *In Vitro* Macrophage Depletion

Approximately 10^7^ MEF cells were trypsinized, washed and resuspended in DMEM without FCS, supplemented with 5 µg of biotinylated α-CD11b antibodies for 30 minutes on ice (or incubated with no antibodies for control groups). Both test and control cells were washed, resuspended in 6 ml DMEM supplemented with 150 µl of Streptavidin-conjugated magnetic beads (Dynal) and incubated at 4°C on a slow moving rotor for 20 minutes. Cells were then placed on a magnetic column (Dynal) for 2 minutes and non-adherent cells were carefully collected. The procedure was repeated twice to increase cell purity, upon which the cells were counted and the efficiency of the procedure was tested on a 10^5^ cell aliquot. Alternatively, macrophages were depleted from MEF preparations using CD11b-conjugated micro beads (Miltenyi Biotec) according to the manufacturer's instructions. The rest of the cells were seeded in 24-well plates at 6×10^5^ cells per well, alone or in combination with macrophages, and used in infection experiments as detailed below.

### *In Vitro* Infection Experiments

Cells were spread on the day before infection in 24-well plates and cells from one representative well were counted in a Neubauer chamber on the day of infection. This typically resulted in a cell count of 60 000 cells per well, hence we infected cells with 2000 PFU in a volume of 400 µl per well to obtain a MOI of 0.03 in our typical infection experiment. Upon 1 hour of incubation, the virus was removed and medium replaced with fresh one. Where indicated, the medium was replaced with medium containing IFNγ (Molecular Probes), Zymosan (Sigma), LPS (Sigma), neutralizing α-TNFα antibodies (Sigma), or z-VAD-fmk (R&D Systems), in the combinations shown for individual experimental conditions. In the supernatant transfer experiment, ANA-I macrophages were infected with indicated virus at a MOI of 1 for 5 days, upon which the supernatant was filtered through a 0.1 µm pore-size filter (Millipore) and transferred to CD11b-depleted MEF cells at 1 hour post infection with the indicated virus.

### *In Vivo* Infection Experiments

Adult male or female mice were infected with purified, tissue-culture derived virus by subcutaneous injection into the hind leg (total volume of 50 µl), by intraperitoneal or intravenous injection (200 µl) or by intranasal inoculation under isoflurane anesthesia (15 µl). At indicated time points after infection, organs were dissected in sterile conditions and stored at −80°C until use for the plaque assay. Macrophage depletion was performed by i.v. and i.p. injections of 200 µl of liposome encapsulated clodronate at 48 and 24 hours prior to viral infection, respectively. Age-matched littermates were treated with liposome encapsulated PBS in the same manner. Depletion of spleen, liver and peritoneal cavity macrophages was confirmed by flow cytometry analysis on a LSRII (BD Biosciences) using the following antibodies: F4/80-FITC, CD11b-PE-Cy7 and Ly6C-APC (Biolegend). NK cell depletion was performed by i.p. injection of 50 µg anti-Asialo-GM1 antibody (eBioscience) 24 hours prior to virus infection. For continued depletion of NK cells, administration of anti-Asialo-GM1 antibody was repeated every seven days. NK cell depletion was confirmed in splenocytes by flow cytometry analysis using NKp46-PE antibody (Biolegend) (data not shown).

### Plaque Assay

Organ homogenates were titrated on MEFs by plaque assay [32] with modifications described previously [16]. In brief, organs were homogenized in 5 ml of DMEM (supplemented with 5% FCS) and diluted in 1∶10 steps. For the determination of *in vivo* titers, the diluted homogenates were layered on MEFs and centrifuged at 1000× g for 30 minutes for enhancement of infectivity, which was followed by 30 minutes of incubation at 37°C. For the titration of virus from experiments performed *in vitro*, the centrifugation step was omitted, but the cells were incubated with the virus for 1 hour at 37°C. In both cases, the supernatants were replaced with an overlay of carboxymethylcellulose (Sigma) to prevent secondary viral spread. Plaques were counted four days later.

### Statistical Analysis

Two experimental groups were compared using the Mann-Whitney test and groups were considered significantly different if the *P* value was <0.05. In comparisons of more than two experimental groups, the Kruskal-Wallis test with Dunn's post-test was applied. *In vitro* titers were compared by student t-test.

## Supporting Information

Figure S1 **ΔM36 MCMV applied subcutaneously is persistent in spleens of immunocompromised mice.** (A) RAG1^−/−^ mice were s.c. infected with 10^5^ PFU of indicated virus and monitored for survival (n = 4–6/group). Mice were sacrificed at day 27 post infection, when the M36rev-infected mice had lost more than 20% of body weight. Infectious virus was determined by plaque assay on MEF cells in spleen, lungs, and salivary glands. (B) Since virus was still detectable in spleens of ΔM36-infected mice at day 27 post infection, new cohorts of RAG1^−/−^ mice were s.c. infected with 10^5^ PFU of indicated virus. At 1, 2 or 3 months post infection infectious virus was determined by plaque assay on MEF cells in spleen. † - M36rev-infected mice died by day 36 post infection. Each symbol represents an individual mouse. Differences in median values are highlighted by grey shading. The dashed line shows the limit of detection.
(TIF)

Figure S2 **NK cells are not responsible for the ΔM36 MCMV growth defect in vivo.** (A) RAGγC^−/−^ mice were s.c. infected with 10^5^ PFU of ΔM36 (○) or M36rev (•) MCMV (n = 5/group) and monitored for weight loss and survival. (B) NK cells were depleted in RAG1^−/−^ mice by i.p. injection of 50 µg anti-Asialo-GM1 antibody. After 24 hours mice were s.c. infected with 10^5^ PFU of ΔM36 (○) or M36rev (•) MCMV (n = 9/group) and monitored for weight loss and survival. γ Mortality also includes mice that were sacrificed because they had lost more than 20% of body weight.
(TIF)

Figure S3 **Macrophages are partially depleted from RAG1^−/−^ mice after treatment with liposome encapsulated clodronate.** RAG1^−/−^ mice received i.v. and i.p. injections of 200 µl liposome encapsulated PBS or clodronate (Cl_2_MBP) at 0 and 24 hours. 24 hours following the second injection, the mice were i.p. injected with 10^5^ PFU of MCMV and 3 days later macrophage depletion was evaluated in (A) spleen, (B) liver, and (C) the peritoneal cavity by flow cytometry analysis using CD11b and F4/80 antibodies to identify macrophages and Ly6C antibody to exclude monocytes. Representative gating of one out of two tested animals per group is shown. Please note that the optimal depletion was achieved in the spleen and peritoneal cavity, but not in the liver. (D) The frequency of F4/80 positive cells (bottom panel of A, B, and C, respectively) is given as percentage of total cells and their reduction upon liposome encapsulated clodronate treatment is expressed as fold change.
(TIF)

Figure S4 **CD11b cells in primary MEF preparations.** Primary MEF cells or NIH-3T3 fibroblasts were trypsinized and stained with anti-CD11b, anti-CD11c (control antibody) or no antibodies. Typical flow-cytometric results are shown as dot blots, where the specific staining is indicated on the y-axis, and the numbers represent the percentage of cells above the indicated threshold line.
(TIF)

Figure S5 **LPS synergizes with IFNγ and inhibits ΔM36 growth to the same extent as Zymosan.** MEF cells were infected with indicated virus in the presence of unsupplemented DMEM (MOCK), or DMEM supplemented with LPS (100 ng/ml) and IFNγ (100 ng/ml) or Zymosan (30 µg/ml) and IFNγ (100 ng/ml). Four days later, infectious virus titer in supernatants was established by plaque assay. Group means + standard deviations are shown.
(TIF)

Figure S6 **The presence of neutralizing TNFα antibodies rescues ΔM36 growth in ANA-I macrophages.** ANA-I (A) or IC-21 (B) macrophages were infected at a MOI of 1 with ΔM36, M36rev or mock-infected either in the presence or absence of neutralizing TNFα antibodies (1 µg/ml). Virus titers in supernatants were determined at day 3 post infection by plaque assay.
(TIF)
