# Supplementary figures and images for: Block of Death-Receptor Apoptosis Protects Mouse Cytomegalovirus from Macrophages and Is a Determinant of Virulence in Immunodeficient Hosts
Source: PLoS Pathog. 2012 Dec 13;8(12):e1003062. doi: 10.1371/journal.ppat.1003062 (PMC3521658; doi:10.1371/journal.ppat.1003062)

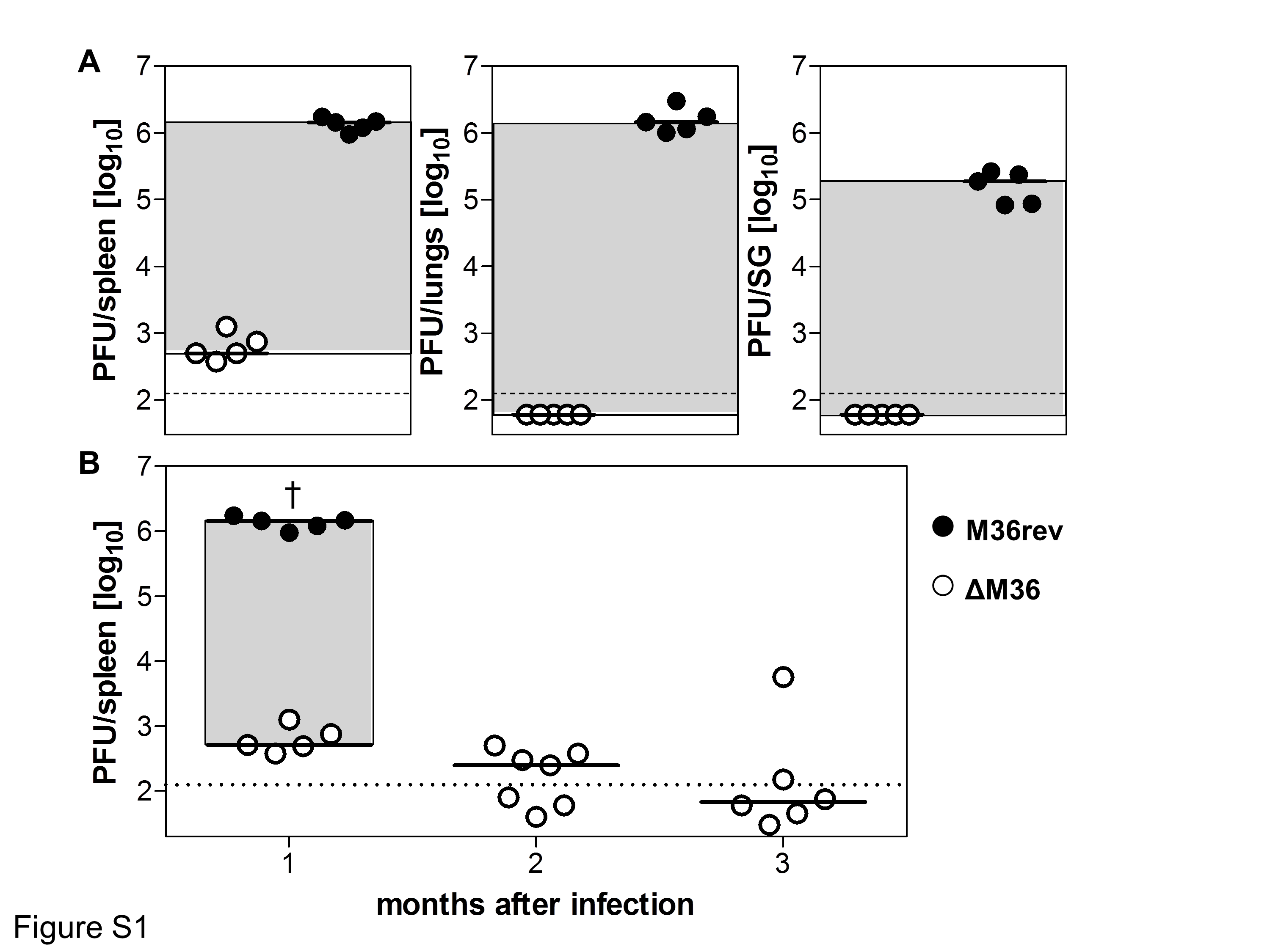

Supplement: Figure S1 — ΔM36 MCMV applied subcutaneously is persistent in spleens of immunocompromised mice. (A) RAG1−/− mice were s.c. infected with 105 PFU of indicated virus and monitored for survival (n = 4–6/group). Mice were sacrificed at day 27 post infection, when the M36rev-infected mice had lost more than 20% of body weight. Infectious virus was determined by plaque assay on MEF cells in spleen, lungs, and salivary glands. (B) Since virus was still detectable in spleens of ΔM36-infected mice at day 27 post infection, new cohorts of RAG1−/− mice were s.c. infected with 105 PFU of indicated virus. At 1, 2 or 3 months post infection infectious virus was determined by plaque assay on MEF cells in spleen. † - M36rev-infected mice died by day 36 post infection. Each symbol represents an individual mouse. Differences in median values are highlighted by grey shading. The dashed line shows the limit of detection. (TIF) [file ppat.1003062.s001.tif]

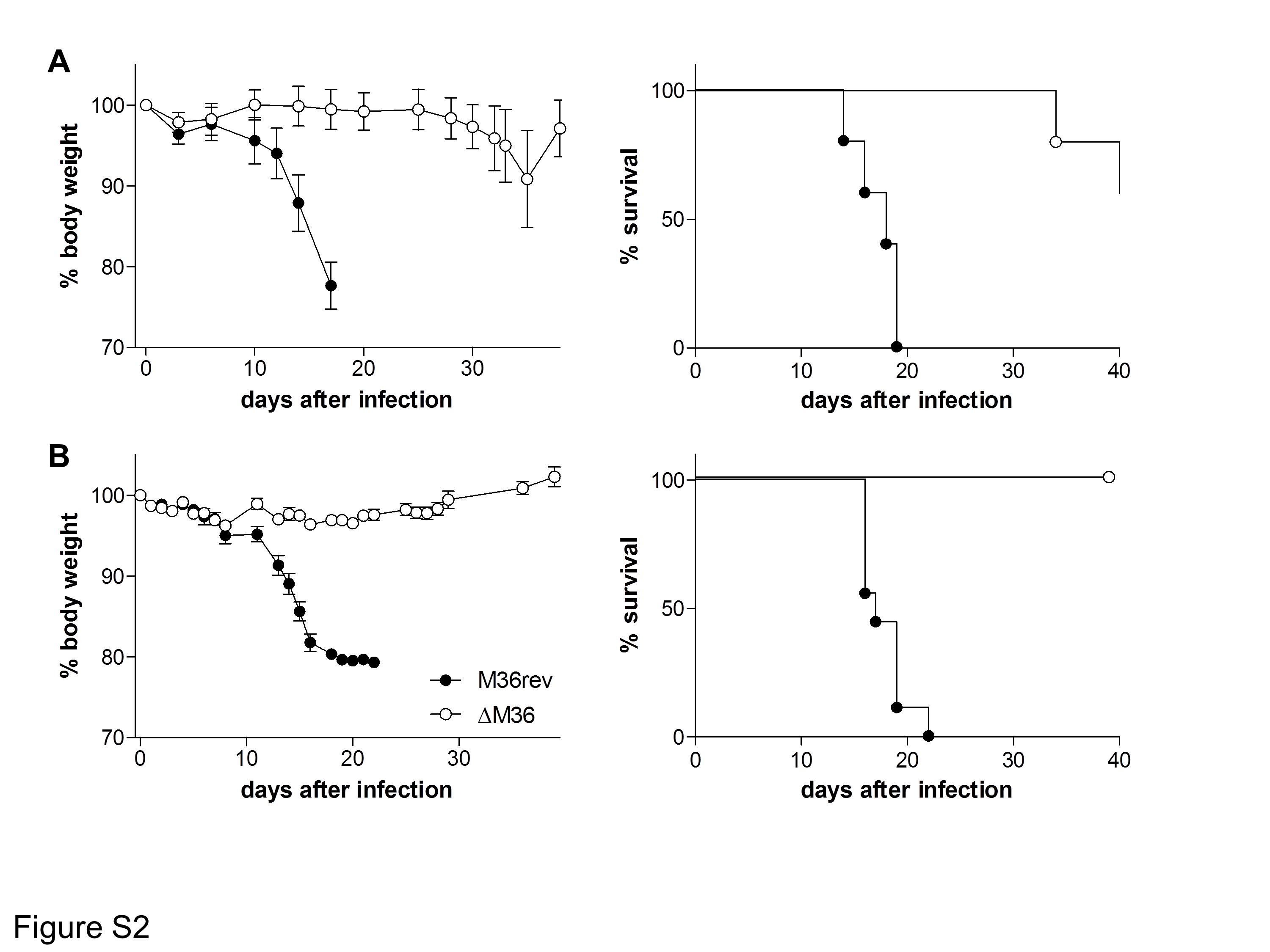

Supplement: Figure S2 — NK cells are not responsible for the ΔM36 MCMV growth defect in vivo. (A) RAGγC−/− mice were s.c. infected with 105 PFU of ΔM36 (○) or M36rev (•) MCMV (n = 5/group) and monitored for weight loss and survival. (B) NK cells were depleted in RAG1−/− mice by i.p. injection of 50 µg anti-Asialo-GM1 antibody. After 24 hours mice were s.c. infected with 105 PFU of ΔM36 (○) or M36rev (•) MCMV (n = 9/group) and monitored for weight loss and survival. γ Mortality also includes mice that were sacrificed because they had lost more than 20% of body weight. (TIF) [file ppat.1003062.s002.tif]

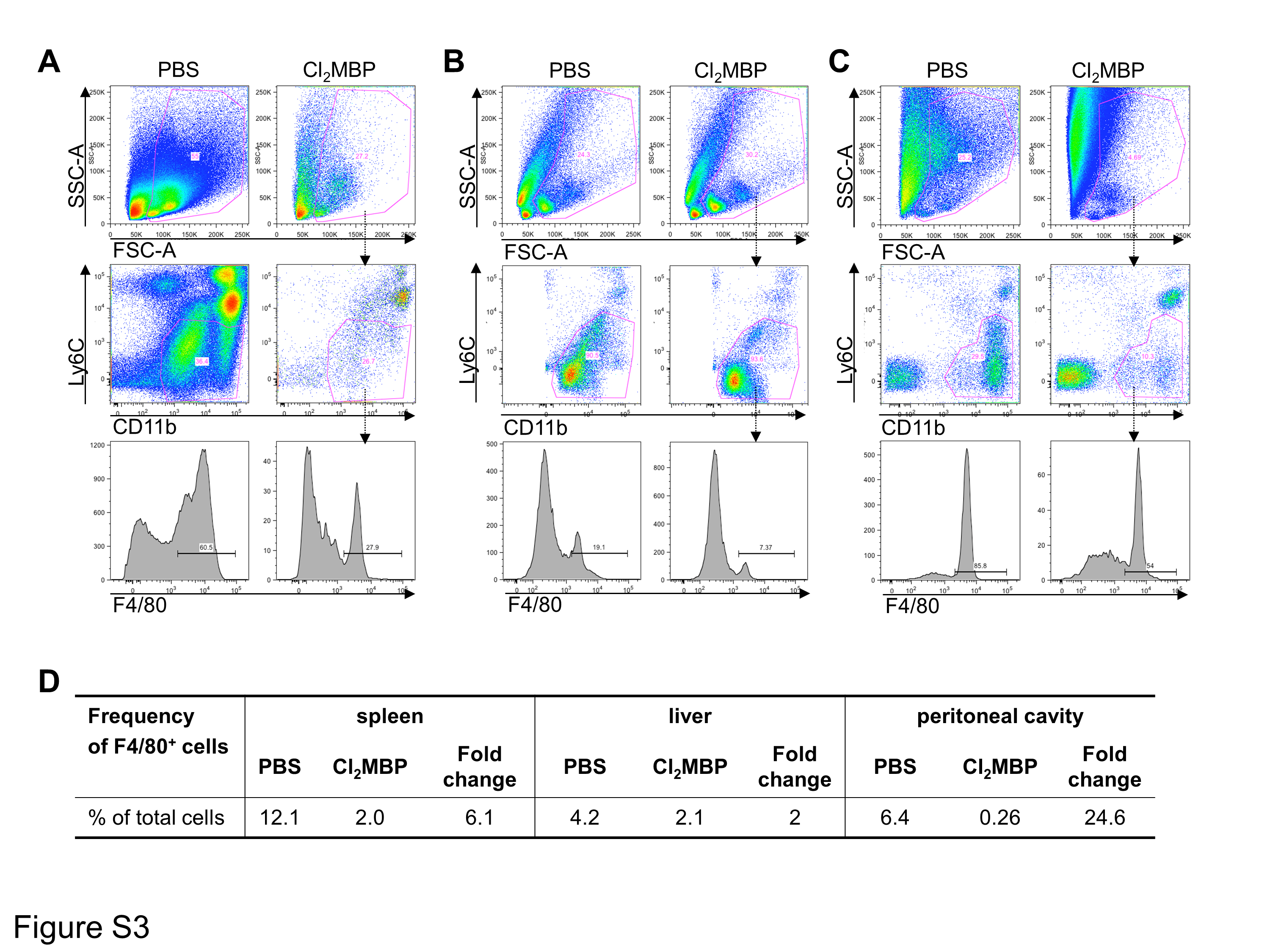

Supplement: Figure S3 — Macrophages are partially depleted from RAG1−/− mice after treatment with liposome encapsulated clodronate. RAG1−/− mice received i.v. and i.p. injections of 200 µl liposome encapsulated PBS or clodronate (Cl2MBP) at 0 and 24 hours. 24 hours following the second injection, the mice were i.p. injected with 105 PFU of MCMV and 3 days later macrophage depletion was evaluated in (A) spleen, (B) liver, and (C) the peritoneal cavity by flow cytometry analysis using CD11b and F4/80 antibodies to identify macrophages and Ly6C antibody to exclude monocytes. Representative gating of one out of two tested animals per group is shown. Please note that the optimal depletion was achieved in the spleen and peritoneal cavity, but not in the liver. (D) The frequency of F4/80 positive cells (bottom panel of A, B, and C, respectively) is given as percentage of total cells and their reduction upon liposome encapsulated clodronate treatment is expressed as fold change. (TIF) [file ppat.1003062.s003.tif]

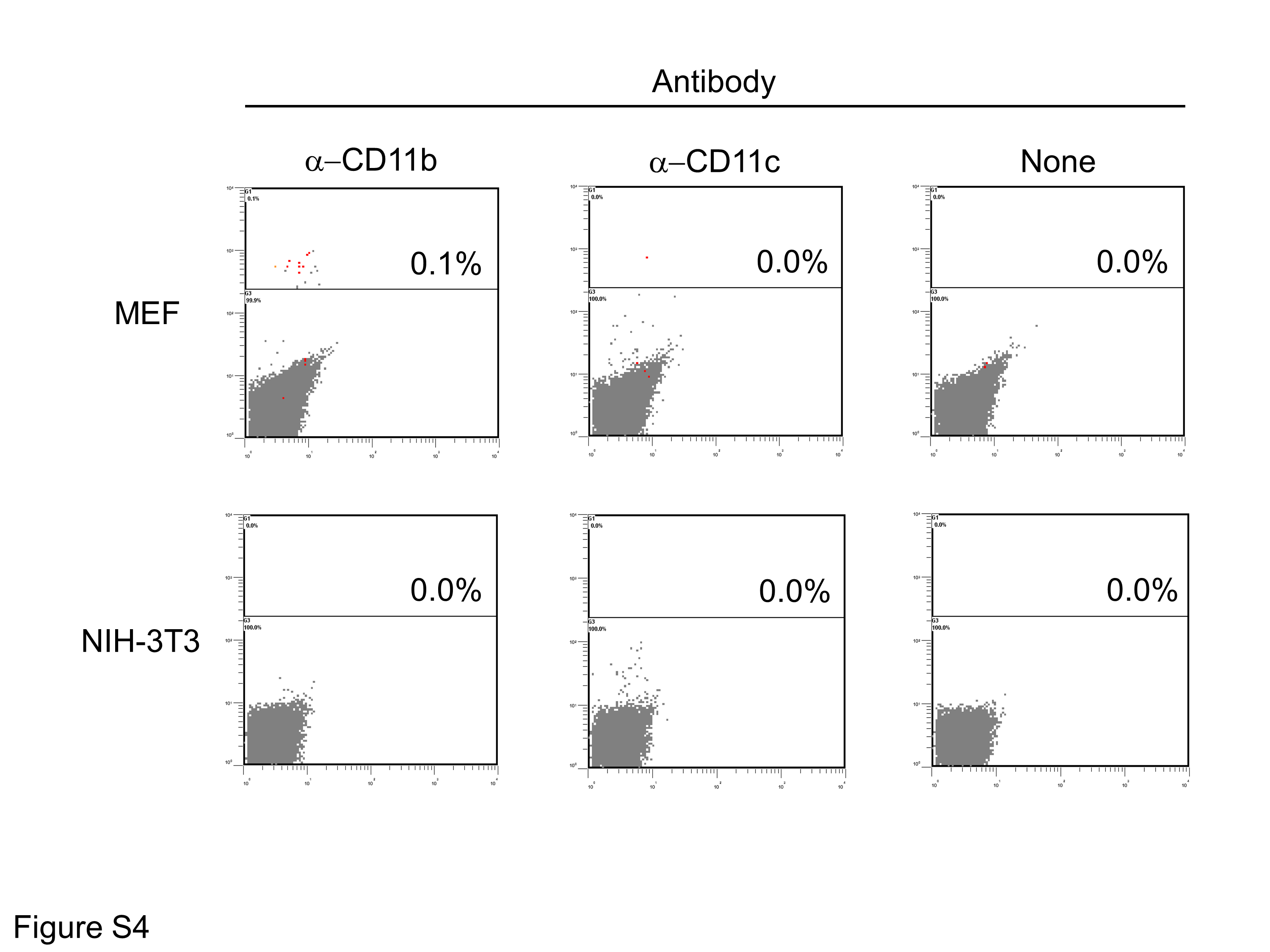

Supplement: Figure S4 — CD11b cells in primary MEF preparations. Primary MEF cells or NIH-3T3 fibroblasts were trypsinized and stained with anti-CD11b, anti-CD11c (control antibody) or no antibodies. Typical flow-cytometric results are shown as dot blots, where the specific staining is indicated on the y-axis, and the numbers represent the percentage of cells above the indicated threshold line. (TIF) [file ppat.1003062.s004.tif]

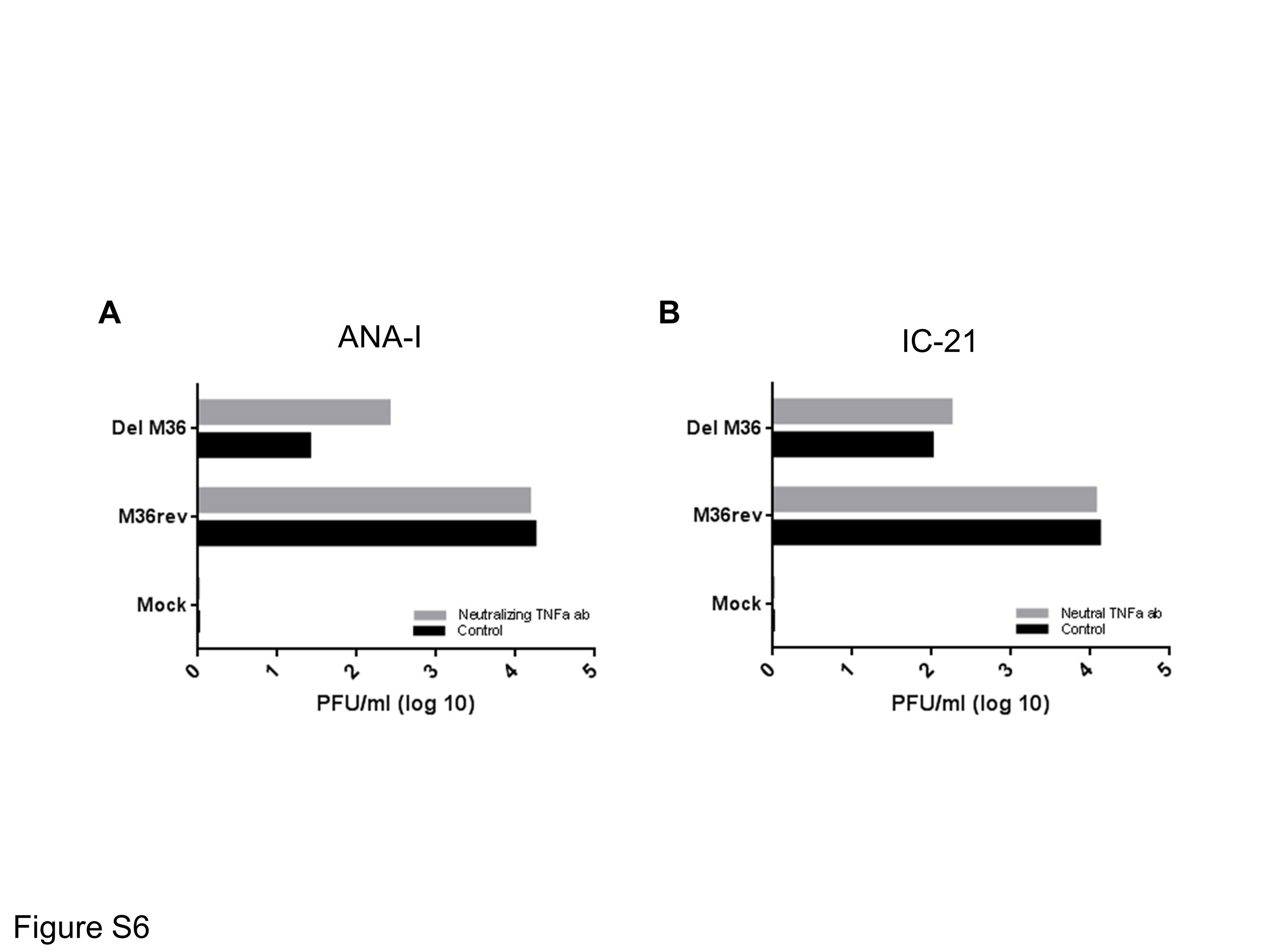

Supplement: Figure S6 — The presence of neutralizing TNFα antibodies rescues ΔM36 growth in ANA-I macrophages. ANA-I (A) or IC-21 (B) macrophages were infected at a MOI of 1 with ΔM36, M36rev or mock-infected either in the presence or absence of neutralizing TNFα antibodies (1 µg/ml). Virus titers in supernatants were determined at day 3 post infection by plaque assay. (TIF) [file ppat.1003062.s006.tif]
